# Supplementary material for: FERN – a Java framework for stochastic simulation and evaluation of reaction networks
Source: BMC Bioinformatics. 2008 Aug 29;9:356. doi: 10.1186/1471-2105-9-356 (PMC2553347; doi:10.1186/1471-2105-9-356)
Supplement: Additional file 1 — FERN distribution, Version 1.3. This archive contains the FERN source code and binaries as well as documentation and example models in FernML and SBML. [file 1471-2105-9-356-S1.zip › fern/doc/javadoc/fern/network/sbml/class-use/SBMLNetwork.html]

Uses of Class fern.network.sbml.SBMLNetwork


---


|  |  |  |
| --- | --- | --- |
| **Overview** \| **Package** \| **Class** \| **Use** \| **Tree** \| **Deprecated** \| **Index** \| **Help** | |  |
| PREV   NEXT | **FRAMES**    **NO FRAMES**     **All Classes** |  |


---


## **Uses of Class fern.network.sbml.SBMLNetwork**

| Packages that use SBMLNetwork | |
| --- | --- |
| **fern.network.sbml** | Provides the classes for parsing and using sbml based networks. |

Uses of SBMLNetwork in fern.network.sbml

Constructors in fern.network.sbml with parameters of type SBMLNetwork

`SBMLEventHandlerObserver(Simulator sim,
SBMLNetwork net,
org.sbml.libsbml.Event event)`
  
          Creates the observer.

`SBMLPropensityCalculator(SBMLNetwork net)`
  
          Creates the `MathTree`s and parses the parameters.

---


|  |  |  |
| --- | --- | --- |
| **Overview** \| **Package** \| **Class** \| **Use** \| **Tree** \| **Deprecated** \| **Index** \| **Help** | |  |
| PREV   NEXT | **FRAMES**    **NO FRAMES**     **All Classes** |  |


---
